# Left-sided facial mass in an adult female

**DOI:** 10.1016/j.radcr.2021.08.059

**Published:** 2021-10-02

**Authors:** Henry Foster, Bradley Schiff, Jacqueline Bello, Keivan Shifteh

**Affiliations:** a Albert Einstein College of Medicine/Montefiore Medical Center, Montefiore Medical Center 111 E 210^th^ St Bronx, NY 10467, USA

**Keywords:** Ameloblastoma, Radiology, Caldwell luc, Mitogen-activated protein kinase, Epigenetic modification

## Abstract

A left maxillary sinus soft tissue mass was discovered on computed tomography in a 70-year-old woman who had been experiencing blood-tinged mucus for 2 years. The lesion demonstrated mild enhancement, and bony destruction. Magnetic resonance imaging displayed a cerebriform appearance of the mass, which mimicked the appearance of inverted papilloma. However, histology and staining identified the lesion as ameloblastoma. Resection of the tumor was successful with no recurrence 1 month later on follow-up computed tomography. This case represents an unusual imaging presentation of ameloblastoma, and an opportunity to avoid the misdiagnosis of inverted papilloma in similar future cases.

## Introduction

Ameloblastoma is a benign tumor of odontogenic origin, but if not treated can cause significant morbidity or death [1,2]. Clinically, Ameloblastoma may be asymptomatic or present with obstructive symptoms [1]. Mandibular ameloblastoma is the most common with only a minority of cases reported in the maxilla [3]. On imaging, Ameloblastoma typically displays a unilocular/multilocular pattern, but demonstrates variable appearances depending on the subtype and presence/absence of aggressive features [1,2]. Dysregulation of cell proliferation is the leading etiology with several identified gene mutations [4]. Epigenetic alterations are an additional emerging etiologic explanation [5]. Surgical excision is the leading treatment, albeit with high recurrence rates [1,2,4]. We present a case of maxillary ameloblastoma with an unusual presentation on imaging which was ultimately diagnosed by histology and staining, and successfully treated with surgery.

## Case report

A 70-year-old woman was incidentally found to have a left maxillary sinus mass on a computed tomography (CT) scan of the facial bones performed for trauma. The patient was referred to Otolaryngology for further workup. She recalled having noticed intermittent blood-tinged mucus for over 2 years with episodes of epistaxis over the last month. She also reported 1 week of left facial numbness, and one day of drooling from her left oral commissure. Her medical history was notable for Human Immunodeficiency Virus, Diabetes Mellitus, Hypertension, and chronic sinusitis. There was no history of tobacco or alcohol use. On physical exam, a palpable mass was noted below the left zygoma, and on endoscopy, the left ostiomeatal complex was compromised secondary to adjacent soft tissue fullness.

**Fig. 1:**
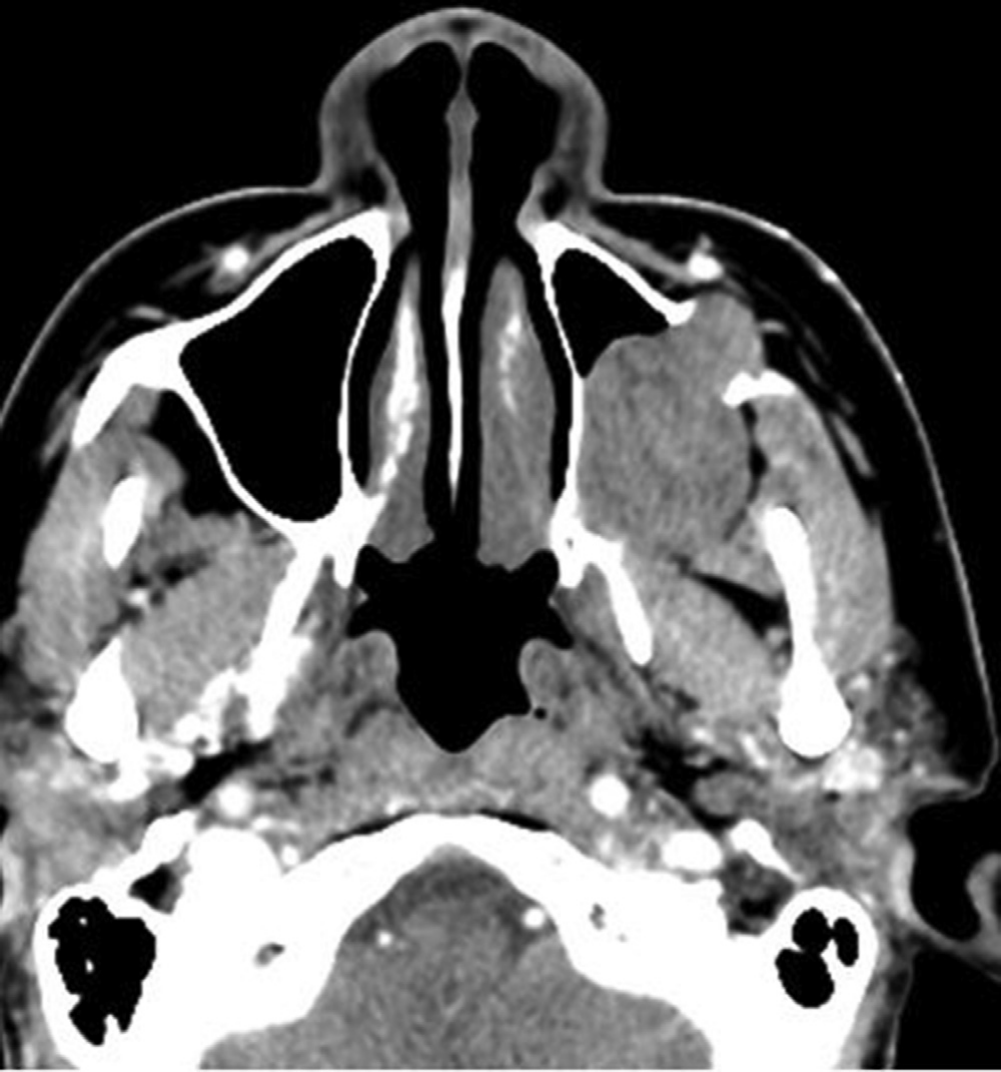
Axial CT post contrast: Soft tissue density mass involving the left maxillary sinus with associated bony erosion of the anterior and lateral walls.

**Fig. 2:**
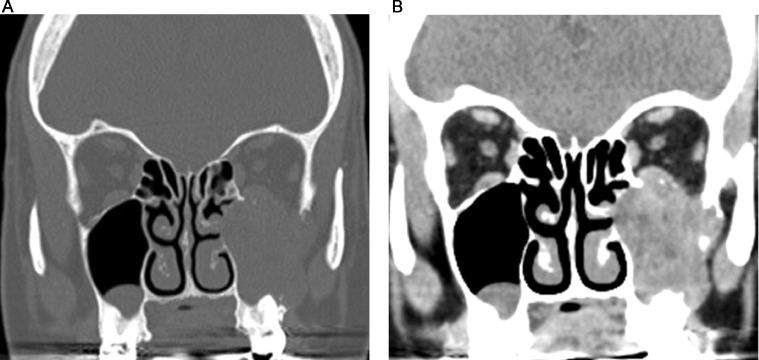
A. Coronal CT bone window: Non-calcified left maxillary sinus mass, with bony erosion along the left orbital floor and lateral wall of the left maxillary sinus, B. Coronal CT soft tissue window: Left maxillary sinus mass, with intra-orbital extension superiorly and buccal space laterally.

**Fig. 3:**
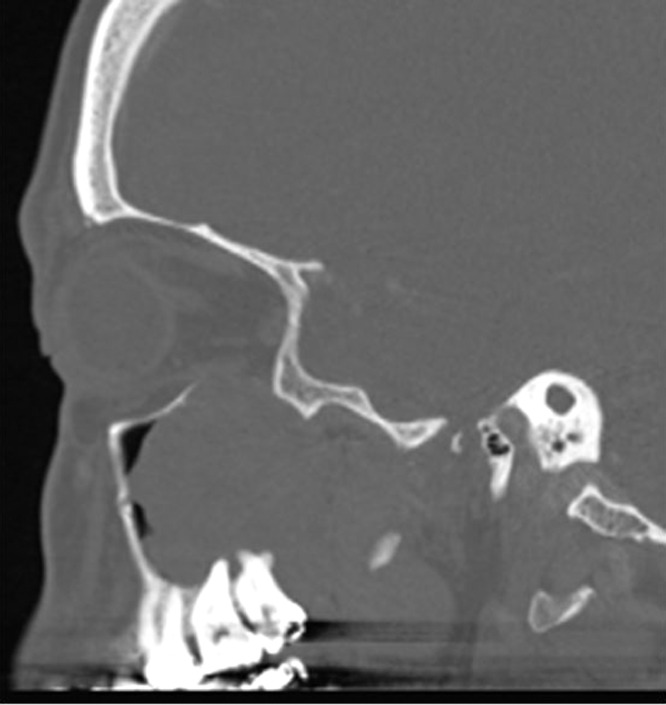
Sagittal CT bone window: The soft tissue mass surrounds the roots of the left second and third maxillary molar teeth without definitive erosion.

**Fig. 4:**
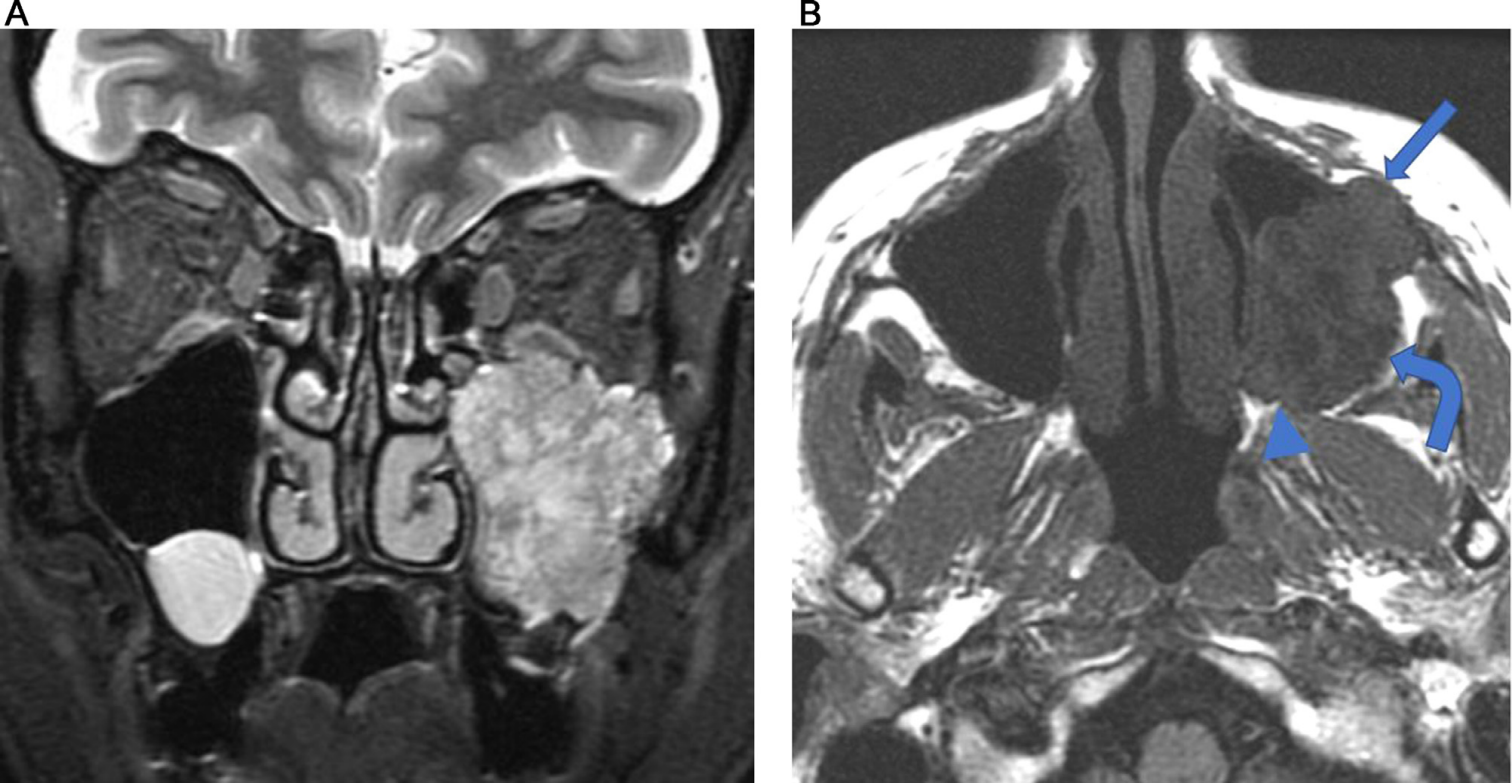
A. Coronal T2 with fat saturation: Heterogeneous mass with cerebriform pattern filling the left maxillary sinus with intra-orbital extension. B. Axial T1 without fat saturation: Soft tissue mass, isointense to muscle, extending anteriorly into the pre-maxillary soft tissue (arrow), laterally into the buccal space (curved arrow), and posteriorly into the pterygopalatine fossa (arrow head).

**Fig. 5:**
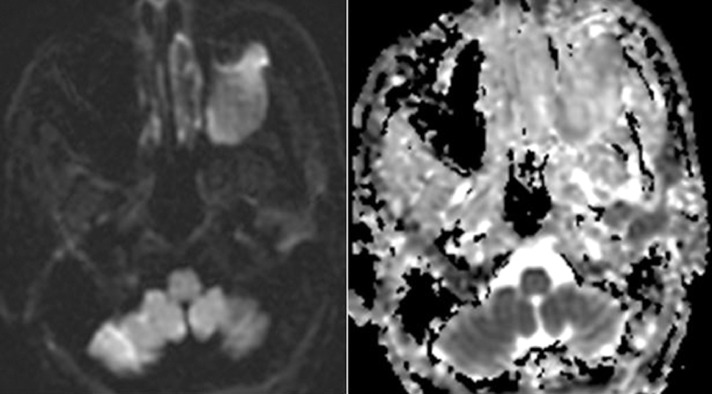
Axial diffusion-weighted images (DWI) and apparent diffusion coefficient (ADC) maps: No restricted diffusion is noted within the left maxillary soft tissue mass.

Contrast enhanced CT of the paranasal sinuses demonstrated a large, soft tissue density mass without significant enhancement in the left maxillary sinus **(**Fig. 1). Bony destruction was noted along the anterior, posterior, and lateral walls of the left maxillary sinus, as well as the left orbital floor (Fig. 2). The soft tissue mass surrounded the roots of the left second and third maxillary molar teeth without definitive erosion (Fig. 3). Non-contrast magnetic resonance imaging (MRI) of the paranasal sinuses showed the soft tissue mass was isointense to muscle on T1 weighted images, extending anteriorly into the pre-maxillary soft tissue, laterally into the buccal space, and posteriorly into the pterygopalatine fossa. On coronal T2 weighted images the soft tissue mass appeared heterogeneous, with a cerebriform pattern, filling the left maxillary sinus with intra-orbital extension and elevation of the inferior rectus muscle (Fig. 4). No restricted diffusion was noted within the soft tissue mass on diffusion-weighted images (DWI) (Fig. 5). Fine needle aspiration of the mass revealed scant groups of cohesive cells with nuclear enlargement, hyperchromasia, and mild cellular crowding, without high-grade features. Special staining further revealed positivity for cytokeratin AE1/AE3, p63, and p40, but was negative for S100, diagnostic for ameloblastoma.

A medial maxillectomy was performed under general anesthesia. Frank tumor was visualized in the maxillary sinus antrum. Resection of the lateral and most inferior aspects of the mass was performed with a Caldwell-Luc procedure, achieving near total resection. Repeat CT 1 month later showed no evidence of recurrence.

## Discussion

Ameloblastomas are locally aggressive benign tumors of the mandible/maxilla of odontogenic epithelium affecting men and women equally at an average age of 35-42. They are five times more common in African Americans compared to Caucasians [1,2]. Maxillary ameloblastoma represents only 15% of all ameloblastomas, and is more clinically aggressive, potentially due to the maxilla's cancellous bone compared to the compact bone of the mandible [3]. Common symptoms of ameloblastoma include a mass and/or swelling, occlusive symptoms, and pain, although 35% of patients are asymptomatic [1]. Epistaxis, as present in this case, is not a commonly reported symptom of mandibular ameloblastoma but has been reported in maxillary ameloblastoma [3].

Ameloblastoma has undergone multiple changes in classification. The 2017 WHO classification system includes 3 types: conventional ameloblastoma, unicystic ameloblastoma, and extraosseous/peripheral ameloblstoma.^6^ The conventional type is the most common, comprising 91% of cases [2]. The unicystic type represents 5%-15% of cases, and is more common in younger patients [2]. The unicystic type is further divided into luminal, intraluminal, and mural types [6]. The desmoplastic type is now a histologic subtype of conventional ameloblastoma due to its lack of significant biological difference [6]. Lastly, the peripheral type includes only 1% of cases, and typically occurs in middle aged patients in the posterior gingiva or alveolar sulcus [2]. Both conventional ameloblastoma and mural unicystic ameloblastoma display more aggressive behavior, resulting in significant morbidity and death if uncontrolled [1,2,6].

Imaging of Ameloblastoma classically demonstrates a “soap bubble-like” appearance, and on CT ameloblastoma presents as a well-defined radiolucent unilocular/multilocular lesion with a radiopaque border [1,2,7]. Aggressive features include erosion of dental roots, cortical destruction, and extraosseous extension [7]. Significant solid portions are found in malignant ameloblastoma [7]. Typical features of Ameloblastoma on MRI include mixed solid and cystic patterns, papillary projections, irregularly thick walls, loculations, and marked septal enhancement on T1-weighted images with gadolinium [8]. MRI plays a vital role in mapping out the extent of disease, including intracranial and intraorbital extension [2]. However in this case the lesion appeared on CT as a non-calcified soft tissue density mass, with a cerebriform appearance on T2 weighted images. These features are atypical for ameloblastoma and are more typical of an inverted papilloma [9].

Histology often shows a follicular/plexiform growth pattern [1,2]. Ameloblastoma with desmoplastic histology displays a mixed radiolucent/radiopaque pattern with irregular borders while the peripheral type demonstrates saucerization [1,2].

The leading etiology of ameloblastoma is Mitogen-activated protein kinase (MAPK) pathway dysregulation, which results in increased cell proliferation [4]. Mutations in the MAPK pathway gene such as BRAF V600E, RAS, and Fibroblast growth factor receptor 2 (FGFR2) have been identified in ameloblastoma [4]. The sonic hedgehog (SHH) pathway has also been found to be altered in ameloblastoma, with a mutated G protein-coupled receptor named smoothened (SMO) being responsible [4]. Over multiple analyses of ameloblastoma cohorts, BRAF V600E mutations have been found to be the most common with indecency ranging from 43%-88% (combined 59% frequency), followed by RAS and FGFR2 combined at 28%, and lastly, by SMO at 14%-39% (combined 22%) [4,6]. BRAF mutations occurred predominantly in mandibular ameloblastoma, and SMO mutations predominantly in maxillary ameloblastoma [4,6].

Epigenetic modifications help to further explain the origin of ameloblastomas. Alterations of DNA methylation, and resulting changes in expression of genes involved in apoptosis, and cell cycle regulation have been associated with ameloblastomas [5]. In addition, long noncoding RNAs influence a wide spectrum of gene expression, and one in particular, KIAA0125, has been associated with ameloblastoma, albeit with unknown function [5]. Overexpression of noncoding RNAs involved in post-translation gene expression, such as MicroRNAs and small nuclear RNAs, has also been demonstrated in ameloblastomas, although with an unclear role [5]. These epigenetic changes may represent targets for future treatments, a means by which to further classify ameloblastomas, or may be used as biomarkers.

Treatment consists of *en bloc* surgical excision with wide bone margin; nonetheless, the risk of recurrence is high in conventional ameloblastoma [1,2,4]. In contrast, both luminal and intraluminal unicystic ameloblastoma have low recurrent rates of less than 10% [4,6]. Peripheral ameloblastoma also has a low recurrence rate [4]. Radiotherapy, with or without chemotherapy, may be considered for recurrent or inoperable tumors [1,2,4]. Future treatment of ameloblastoma may include drugs targeting the BRAF, FGFR2, or other MAPK mutations, although this is currently confined to in vitro studies and a limited number of case reports [4].

## Informed consent

The patient provided written informed consent regarding the publication of this case and the accompanying radiographic images. No identifiable patient information was included in the manuscript.
